# Tox-Database.net: a curated resource for data describing chemical triggered in vitro cardiac ion channels inhibition

**DOI:** 10.1186/2050-6511-13-6

**Published:** 2012-08-13

**Authors:** Sebastian Polak, Barbara Wiśniowska, Anna Glinka, Miłosz Polak

**Affiliations:** 1 Unit of Pharmacoepidemiology and Pharmacoeconomics, Faculty of Pharmacy Jagiellonian University Medical College, Medyczna 9 Street, 30-688, Krakow, Poland

**Keywords:** In vitro data, Cardiotoxicity, Sodium current, Slow potassium current, Rapid potassium current, L-type calcium current, IC50

## Abstract

**Background:**

Drugs safety issues are now recognized as being factors generating the most reasons for drug withdrawals at various levels of development and at the post-approval stage. Among them cardiotoxicity remains the main reason, despite the substantial effort put into in vitro and in vivo testing, with the main focus put on hERG channel inhibition as the hypothesized surrogate of drug proarrhythmic potency. The large interest in the IKr current has resulted in the development of predictive tools and informative databases describing a drug's susceptibility to interactions with the hERG channel, although there are no similar, publicly available sets of data describing other ionic currents driven by the human cardiomyocyte ionic channels, which are recognized as an overlooked drug safety target.

**Discussion:**

The aim of this database development and publication was to provide a scientifically useful, easily usable and clearly verifiable set of information describing not only IKr (hERG), but also other human cardiomyocyte specific ionic channels inhibition data (IKs, INa, ICa).

**Summary:**

The broad range of data (chemical space and in vitro settings) and the easy to use user interface makes tox-database.net a useful tool for interested scientists.

**Database URL:**

http://tox-database.net.

## Background

Drug development is a time and resource consuming process with multiple potential obstacles. Until very recently pharmacokinetic and biopharmaceutical issues were recognized as being the major obstacles, although due to new approaches and tools they were mostly bypassed [1]. Drug safety and toxicity issues are now recognized as being factors generating the most reasons for drug withdrawals at various levels of development and at the post-approval stage. The latter one remains especially harmful as the costs include not only financial, but also a population health burden [2,3]. In recent years much regulatory, industry and public attention has been focused especially on cardiotoxicity. This due to the leading position of cardiotoxicity among the reasons for drug withdrawals, relabeling and late attritions. QT prolongation and potentially fatal arrhythmia, torsade de pointes, occurrence are the most pronounced manifestations of a drugs’ cardiac toxic effects, thus evaluation of the proarrhythmic potential of an investigated compounds is now an integral element of the safety profile required for the approval of new drugs. For all drugs with a proven proarrhythmic activity the capacity to inhibit the rapid delayed rectifier potassium current, IKr, carried by the membrane channel which is composed of subunits encoded by the human ether-à-go-go related gene (hERG) was recognized as being an underlying mechanism of such an effect. However, it has been suggested that the hERG blocking potency, defined as a compound concentration producing 50% inhibition of the IKr current, is an imperfect surrogate marker of the proarrythmic potential of the tested substance. There is no straight correlation between the extent of QT prolongation and TdP occurrence probability, what is more QT prolongation seldom leads to TdP. This can be the result of the concomitant inhibition of other channels, sarcomers contractility modification, alteration of sympatethic heart control or others. It shows that the clinical effect of drugs which influence heart activity is a complex process, which should be considered during early cardiac safety assessment.

The pharmaceutical industry, which is operating in a scientifically vibrant and extremely dynamic environment, is constantly looking for new R&D approaches. Their aim would be to stabilize the success-to-failure ratio regarding the currently investigated compounds. The above mentioned novel approaches include for example the wide incorporation of modelling and simulation tools (PBPK, PK-PD, IVIVE). Apart from such incorporation methods, sharing knowledge via publicly available databases is recognized as a necessary element of the improvements in drug toxicity testing, which has been postulated in Toxicity Testing in the 21st Century: A Vision and a Strategy, and other sources [4,5]. However, this still remains an overlooked instrument with underestimated potential gains, especially in the highly competitive market. There are, although, multiple examples where knowledge dissemination and sharing has been recognized as an essential element of further scientific and practical development. The most significant examples of such initiatives are listed below.

OCHEM is an online database of various experimental measurements for a wide range of chemicals, integrated with the modelling environment. Created as an effect of multinational cooperation under the EU project umbrella. According to the presented information, OCHEM contains 502 313 experimental records for about 411 properties, collected from 4 704 sources. Its uniqueness results from the philosophy of development, as it works under Wiki-style principles and users are encouraged to submit data and correct the inaccurate data. OCHEM contains 4 separate subsets with data describing hERG channel inhibition with various endpoints (EC50, IC50, Ki and percentage of channel blocking) and a different number of records (up to 2 264). For some of them, the basic experimental settings are presented (cell line, temperature maintained during the study) [6].

ACToR is the EPA's online warehouse, granting access to publicly available chemical toxicity data. As granted by the EPA this tool aggregates data from over 500 public sources, on over 500 000 environmental chemicals, which are searchable by chemical name and chemical structure. Specific toxicity databases include ToxRefDB, ToxCastDB, ExpoCastDB and DSSTox, which contain data characterized by various endpoints and research methods. There is a high level of convergence with the NCBI tools for flexible search and navigation. There are no specific subsets offering information regarding the drug triggered hERG channel inhibition [7].

hERGAPDbase is a database of electrophysiological experimental data, documenting potential hERG channel inhibitory actions and the APD-prolongation activities of chemical compounds. All data entries were manually collected from scientific papers. The database enables free access to the electrophysiological experimental data on chemical compounds [8].

hERGCentral powered by Johns Hopkins Ion Channel Center (JHICC), offers access to a library of over 300 000 diverse compounds characterized by an affinity to the hERG channel, although some records are empty. The data is mainly derived from electrophysiological assay using the population patch clamp mode on the Ionworks Quattro, but information from scientific literature sources and online reports and chemical library collections are also incorporated. Queries can by based either on the chemical structure or the properties of a compound and on activity of interests, e.g., IC50 and tail current inhibition [9].

The tox-portal.net service offers two datasets compiled previously for publication needs (offered as supplementary materials). The datasets include IKr and IKs inhibition data in the flat form of an MS Excel file without any search and display capabilities [10,11].

The above presented initiatives are mainly driven by academic and governmental institutions, but the recent years have also brought some examples of close scientific cooperation between direct competitors regarding scientific research, such as the IMI, to name just one of them [12].

These data sources contain diverse information about a compounds’ effects on heart electrophysiology, however, there is in general lack of comprehensive descriptions of in vitro experiments. It is likely that the manual patch clamp technique, which is considered as the gold standard for drug-hERG interaction assessment was used although there is no detailed information provided. What is more, none of those sources contain detailed information about other than hERG and less often discussed in the literature channels, yet they remain very important from a drug safety analysis point of view. Chemical driven inhibition of the cardiomyocyte L-type calcium and sodium channels plays a vital role in the clinically observed outputs of the drug driven changes in ECG. For that reason one of the aims of the work was to deliver comprehensive information describing chemicals and non-potassium channels interplay.

### Construction and content

Tox-database.net is a tool developed for the dissemination of information on early drug safety testing. Its focus is put on in vitro cardiotoxicity assessment.

### Data collection

The previously collected and described sets of data were a starting point for the current work. The collection of the data describing the inhibiting activity of the hERG channel blockers, contained information about 263 molecules described in 642 records [10]. The dataset was reduced to studies carried out on three main cell models (XO, CHO, HEK) and contains description of the in vitro studies settings. Chemicals inhibiting the IKs current were described in the recently published study [11]. For both studies, a similar methodology of data collection was applied, and with no further changes utilized for the current study. Scopus, Medline and Google Scholar searches were performed. There was no time-limit for the search query. In the first step the key phrases were: ‘{short name of the current}’, or ‘{full name of the current}’, or ‘{ion name} channel’, or ‘{ion name} current’ or '{ion channel abbreviation}’ or ‘{ion current abbreviation}’ and IC50 either in the article title, keywords or abstract. If there weren’t any results available for the combination of a compound name with any of the keywords, then the compound’s class name was used in the query.

Every available English-language paper was carefully evaluated. As the experimental IC50 values were obtained originally by the authors of the publication, the results were noted down in the prepared Excel spread sheet. Otherwise, the cross-references were retrieved, if feasible. In addition to the half-maximal inhibitory concentrations (the IC50 values), papers were revised for additional information as follows:

· cellular model applied in experiments,

· channel subunits expressed in the cellular model,

· type of transfection,

· measurement technique,

· experimental conditions (temperature and K^+^ concentration in external solution),

· voltage protocol (protocol type: step, ramp, step-ramp; holding potential, depolarization potential, measurement potential; depolarization pulse time),

· stimulation frequency

· inhibition potential for the defined chemicals concentration.

Our aim was to collect a full set of information for all available chemicals, but it was not always possible. The reasons differed from each other, but in most cases some values were not reported in the literature source. Despite the incompleteness of some of the records, we decided to include all of them in the database to give the widest possible picture of the in vitro measured currents inhibition studies. In such situations either free space was left or in cases of uncertain information, the location in the source paper was noted.

The quality and reliability of the gathered data is an essential element. According to this, strict quality control procedures were implemented at the collection level. All authors involved in acquiring the information are responsible for the control of the other person’s work. Acquire – control pairs were assigned randomly to avoid bias. In cases when the discrepancy between information presented on figures and reported in the main text were observed, authors were contacted and asked for further explanation.

### Description of the collected data

Information describing the results of the in vitro studies, investigating the chemicals-ionic currents interactions, has been collected from publicly available literature sources. The four main human cardiomyocytes ionic currents have their representation - IKr (rapid delayed rectifier potassium current), IKs (slow delayed rectifier potassium current), INa (fast sodium current), ICaL (L-type calcium current) as described in Table 1.

**Table 1:** Characterization of the four ionic currents present in the Tox-Database.net

**Ion**	**Type**	**Current**	**Phase**	**Channel**	**Gene**	**Subunit**	**Mutations and polymorphisms**	**Disorders associated with mutations**	**Source**
Na^+^	Voltage-gated	INa (fast sodium current)	0	Nav1.5	SCN5A	alfa	H558R, S216L, del AL 586-587, R680H, R1193Q, T1304M, F1486L, V1951L, F2004L P2006A, S1103Y, R190G, A572D	- Long QT syndrome type 3	[13-25]
								- Sudden cardiac death	
								- Risk Factor for Atrial Fibrillation	
								- Brugada syndrome	
								- Idiopathic ventricular fibrillation	
								- Heart rhythm disorders	
								- Romano-Ward syndrome	
								- SIDS (Sudden infant death syndrome)	
Ca ^2+^	Voltage-gated	ICaL (L-type calcium current)	2	CaV1.2	CACNA1C	alfa 1 C	G406R, G402S	- Timothy syndrome	[19,22,26,27]
K^+^	Voltage-gated	IKr (rapid delayed rectifier potassium current)	3	Kv11.1	KCNH2 (hERG)	alfa	P347S, R1047L, A1116V, K897T, P967L, Q1068R, R181Q, G187S, GAG187-189del, A190T, A203T, N257H, T367S, G873S, P910L, R1035W, A1058E, N33T, R176W, V215G, H254Q, C723R, P917L, L1023del, A915V, P251A, G965R, R1055Q, L1108V, G1154S, T875M, R273Q, V279M, R885C, S1040G, G294V, A190T, N588K	- Long QT syndrome type 2	[13,14,17-20,22,23,28-37]
								- Romano-Ward syndrome	
								- SIDS	
								- Short QT syndrome	
				MiRP1	KCNE2	beta	Q9E, A66V, T8A, R27C	- Long QT syndrome type 6	[13,14,17-20,22,23,25,28,31,33,36,38]
								- Familial atrial fibrillation	
								- Romano-Ward syndrome	
								- SIDS	
		IKs (slow delayed rectifier potassium current)	3	KvLQT1 (Kv7.1)	KCNQ1	alfa	IAP54-56dup, V129I, V207M, G297S, F335L, P408A, P448R, R451Q, G621S, G643S, V648I, V110I, K393N, D428G, R519H, P441S, G119D, I274V, G460S, V307L, T600M, V648I	- Long QT syndrome type 1	[13,14,17,19,20,23,31,39,40]
								- Atrial fibrillation	
								- Jervell and Lange-Nielsen syndrome - Romano-Ward syndrome	
								- SIDS	
				MinK	KCNE1	beta	G38S, G52A, K69R, D85N, V109I, V14I	- Long QT syndrome type 5	[13,14,17-20,22,23,31,34,36,41]
								- Jervell and Lange-Nielsen syndrome	
								- Romano-Ward syndrome	

The final list of publications consists of 362 positions, which refer to 419 different molecules connected with the above listed currents inhibition (753 - IKr, 165 - IKs, 177 - INa, 181 - ICaL). All records are described by the in vitro research settings and catalogued by their IUPAC names and canonical smiles with links to the original publication (PMID). The latter one utilizes the PubMed classification system with a dynamically created link, based on the PMID number, uploaded in the dataset file. The database is freely available after registration on the http://www.tox-database.net portal [42].

There is a varied range of data presented for the different currents, depending on the in vitro research settings. Table 2 contains a description of the information for all four ionic currents having their representation in the tox-database.net collection.

**Table 2:** Description of information collected for all ionic currents

**Parameter name [unit]**	**Ionic current**				
	**ICa**	**IKr**	**IKs**	**INa**	**Description**
channel type	+	-	+	+	For the cell lines heterologously expressing channel subunits: name of the gene encoding expressed subunits or name of expressed channel and its origin (r-rat; h-human)
in vitro cell model	+	+	+	+	Cell line used for channel subunits expression or cardiomyocytes origin (e.g. HEK, XO, guinea pig VM)
stimulation frequency [Hz]	+	-	-	+	Number of current provoking pulses delivered every second expressed in Hz
transfection type	+	+	+	+	Mode of channel expression in heterologous system (stable or transient)
temperature [^0^C]	+	+	+	+	Temperature maintained during experiment
technique	+	+	+	+	Technique used for current recordings
Ca2+ bath [mM]	+	-	-	-	Calcium ion concentration in the bath solution
K+ bath [mM]	-	+	+	-	Potassium ion concentration in the bath solution
Na+ bath [mM]	-	-	-	+	Sodium ion concentration in the bath solution
t1 pulse [ms]	+	+	+	+	Duration of first pulse in the step-protocol
t2 pulse [ms]	+	+	+	+	Duration of second pulse in the step-protocol
holding potential [mV]	+	+	+	+	The starting membrane voltage level
depolarization potential [mV]	+	+	+	+	Voltage of membrane depolarizing impulse
repolarization potential [mV]	+	+	+	+	Voltage of membrane repolarizing impulse
measurement potential [mV]	+	+	+	+	Membrane voltage level during current amplitude measurement
protocol	+	+	+	+	Type of voltage protocol applied to elicit current (e.g. step, AP, ramp)
data source	+	+	+	+	First author name and publication year
PMID	+	+	+	+	PubMed Unique Identifier, a unique number assigned to each PubMed record
IC50 [μM]	+	+	+	+	The concentration of compound that results in 50% inhibition of current in micromolar
comments	+	+	+	+	Additional information e.g. alternative naming, denotation of smiles and/or IUPAC name source if not PubChem; source of uncertainty

Where: "+” – parameter present in the record description; "-” – parameter absent in the record description.

As it was mentioned above we aimed to collect a full set of information for all available chemicals. Incompleteness of the data presented in the original sources is a limiting factor, and due to this it was not always possible. Additionally, in some cases the published information was questioned at the verification stage. In all such situations the questionable or unclear bits of information were clearly marked with a question mark, which is displayed in the result tables. Further investigation of them can be done by the user, based on data source proofing. An example of such a case is caffeine where no IC50 value was established, although the data points describing the IKr current inhibition for certain concentrations were presented.

### Database structure and technology utilized

The tox-database.net service was written in the Struts2 web application, with the JSP servlet classes supported by the struts-tags. It has been deployed on the Resin server container, shared with the main tox-portal.net application. The service database runs on the MySQL server which is shared with all other tox-* services.

The database is managed from a dedicated control panel, that allows for easy updating of the records in a unified format directly from file. The administrator obtains freedom in selecting applications for formatting (i.e. text editors, MS Excel, OO Calc, LO Calc), the application ensures consistency and optimization of the database entries. In the process of updating, certain records are updated and sent to the relevant base relations. The Management Panel was launched as a web application, available under the authentication of all tox-* services (tox-portal.net, tox-comp.net, tox-database.net).

**Figure 1:**
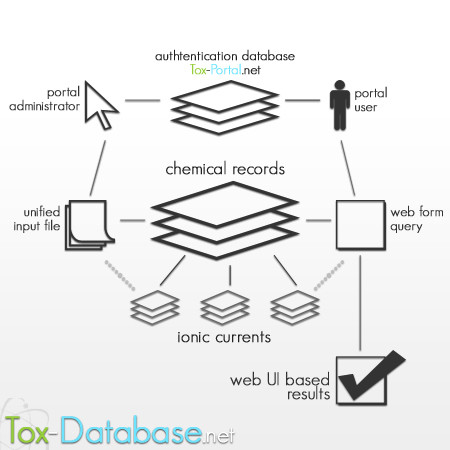
Tox-Database.net management scheme.

**Figure 2:**
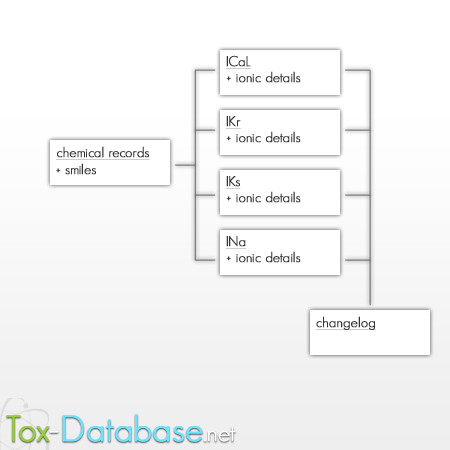
Tox-Database.net presentation layer structure.

The public search form allows for preparation queries to the chemical records' database. Record listing requires authentication approval, based on the users' records collected and stored in the general database authentication that are shared with other tox-* tools. The interface is available in a fully-web manner application. The presentation layer is based on html/css documents. The JavaScript language extended to the popular jQuery library is responsible for the interface operations (Figure 1, Figure 2).

## Utility and discussion

### Web interface

**Figure 3:**
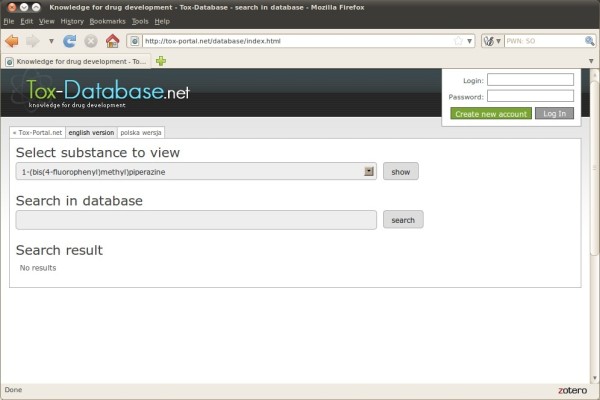
Tox-Database.net GUI – welcome screen.

**Figure 4:**
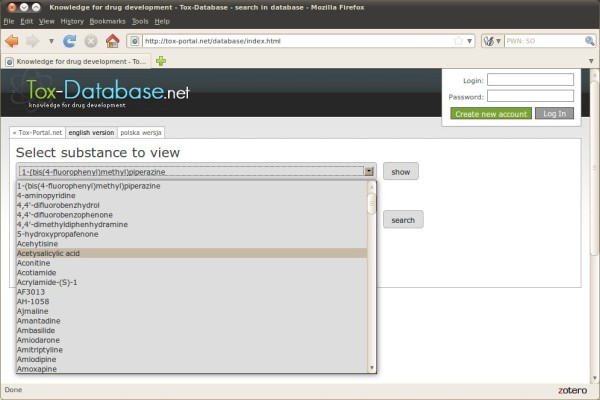
Tox-Database.net GUI – listing mode.

It was decided to use the internet as the only dissemination media, and consequently no off line version is available (Figure 3). There were two assumptions underlying the user interface development - integration with the existing tools, namely the tox-portal.net and tox-comp.net and user friendliness. The first one has been established by the direct utilization of the existing users' accounts registration and maintenance engine. The latter one, included both an intuitive search interface and results display. There are two modes of use which differ from each other. In the first option the user can only browse the list of the collected chemicals sorted alphabetically and no other activities are allowed. There is no need to register an account and log in for such an activity activity (Figure 4).

Account registration is needed to have full access to the database's browsing capability, search tool and results display. The account needs to be activated by the tox-portal.net portal administrator to avoid fake account registration and robots. Straight after the activation fully free access is granted.

### Database usability - search and results display

There are two main types of use after log in. The browsing capability allows for the listing of the alphabetically sorted chemicals which build the database (*Select substance to view*) and the following results display. The *Search in database* option allows for the flexible exploration of the database. There are three search domains:

· canonical smiles

· international names

· IUPAC names

**Figure 5:**
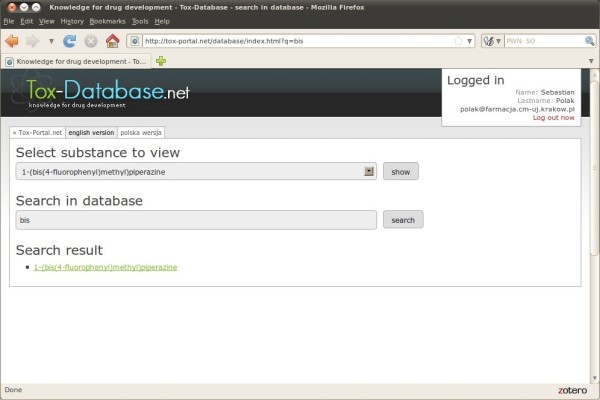
Tox-Database.net GUI – search by name.

**Figure 6:**
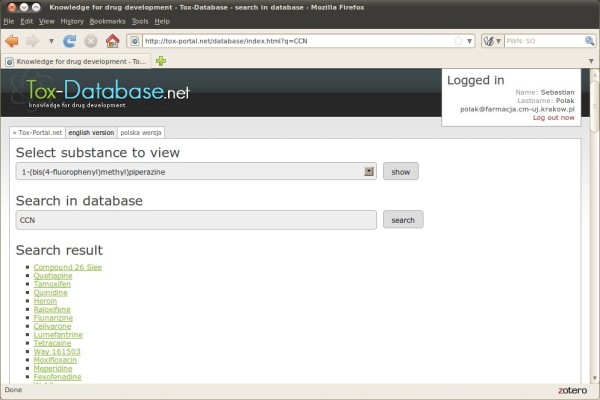
Tox-Database.net GUI – search by smiles.

A minimum of three characters needs to be written down in order to run the search engine. The system displays a list of records filling the previously defined requirements. The search system is sensitive to the typed characters regardless of the search domain. Figure 5 presents 1 output found in the IUPAC name and Figure 6 respectively presents the records found in the canonical smiles strings after typing 3 characters (bis and CCN respectively).

**Figure 7:**
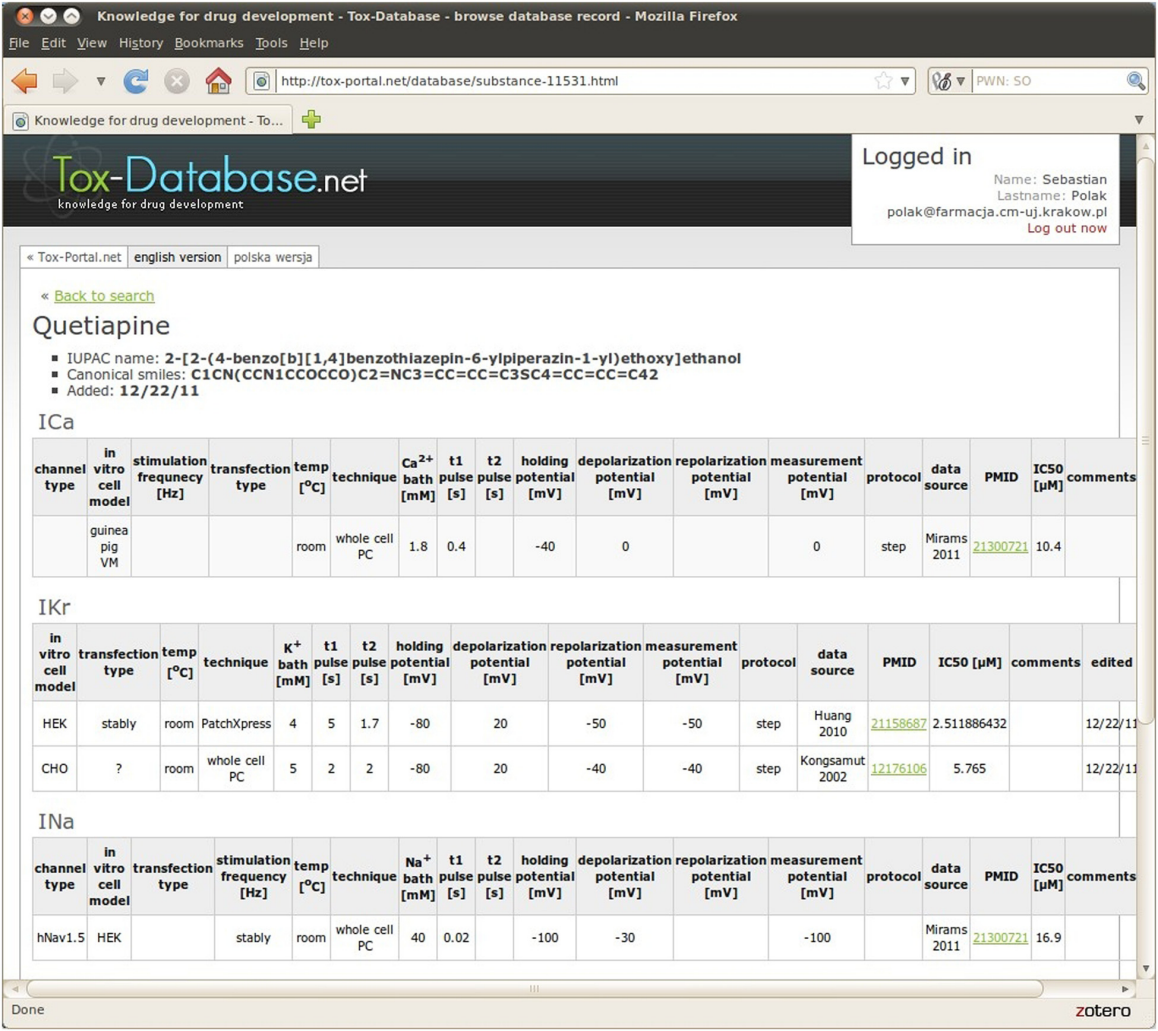
Tox-Database.net GUI – results presentation; multiple channels data.

The headline of the web page, with the individual record displayed, contains the international name, the IUPAC name, canonical smiles (the latter two mainly after the official PubMed data) and information about the publication date.

The detailed results have a table format with the clearly marked ionic current, which has been modified by the chemical of interest. If there is data available for more than one ionic current the tables are displayed in alphabetical order, after the current name.

### Intended use

The drug cardiotoxic effect, with the mechanism based on the direct perturbation of the electrophysiological processes, is one of the most widely described and discussed issues. It is mainly connected with the hERG channels blockade and the following IKr current inhibition, that has been addressed in the regulatory guidelines, which suggests the detailed study of such phenomena on various in vitro models. Additionally other in vitro cellular models as well as ex vivo and in vivo animal models are utilized for the general proarrhythmic potency assessment at the higher level of complexity [19]. Although hERG channel inhibition is a widely accepted surrogate of clinical TdP risk, it is by no means perfect. There are drugs that are hERG inhibitors but do not cause TdP, which may also occur in patients treated with weak in vitro hERG inhibitors. It was hypothesized that drugs, simultaneously influencing other channels (potassium, calcium, sodium), can cause either amplification or reduction of the torsadogenic effect. For that reason the multichannel interaction of the tested compounds should be taken into consideration, in order to make the proarrhythmic risk assessment more reliable [43,44]. Wide in vitro screening for the channels affinity is suggested regardless of the further steps undertaken. For the biophysically-detailed models of cardiac electrophysiology based simulations such information allows for a more reliable prediction of the potential drugs proarrhytmic activity as suggested by the ‘state-of-the art’ article published recently [45]. For situations when no *in vitro* data is available *in silico* predictions can be used. Presented in the tox-database.net collection can be directly used for QSAR models development [11,46].

The aim of the database development and publication was to provide a scientifically useful, easily usable and clearly verifiable set of information describing not only IKr (hERG), but also other human cardiomyocyte specific ionic channels inhibition data.

## Conclusions and further development plans

To the best of our knowledge, the presented database is the only publicly available source of data presenting quantitative information describing the interaction between chemicals and the in vitro realized ICaL/INa cardiac ionic currents. Additionally, wide sets of data describing similar interaction for the potassium currents (IKr/IKs) have been published. The user friendly interface allows for easy search and browsing. Tox-database.net is freely available after registration.

Further development plans include two parallel paths. The first one is mainly focused on a further increase of the number of records and catalogued chemicals in the existing database. The only source of information remains the peer-reviewed, publicly available articles published in scientific journals. It is also planned to increase the number of the ionic currents possibly altered by drugs and other chemicals regardless of the inhibition effects (pro- or antiarrhythmic). The literature analysis indicates that the possible targets are Ito (distinct transient outward potassium current) and IK1 (native inward rectifier potassium current). Both mentioned channels have been relatively recently discovered and the number of inhibition studies carried out is limited.

## Availability and requirements

The described database is available at http://www.tox-database.net. There are no restrictions for commercial and non-commercial use.

## Competing interests

The authors declare that they have no competing interests.

## Authors' contributions

SP conceived and coordinated the study, participated in data gathering and quality control, drafted the manuscript. BW participated in data gathering and quality control, drafted the manuscript. AG participated in data gathering and quality control. MP designed the database structure and prepared GUI for the database administrators as well as web display part. All authors read and approved the final manuscript.

## Pre-publication history

The pre-publication history for this paper can be accessed here:

http://www.biomedcentral.com/2050-6511/13/6/prepub
